# Binding Affinity of Trastuzumab and Pertuzumab Monoclonal Antibodies to Extracellular HER2 Domain †

**DOI:** 10.3390/ijms241512031

**Published:** 2023-07-27

**Authors:** Victor L. Cruz, Virginia Souza-Egipsy, María Gion, José Pérez-García, Javier Cortes, Javier Ramos, Juan F. Vega

**Affiliations:** 1 BIOPHYM, Department of Macromolecular Physics, Instituto de Estructura de la Materia, IEM-CSIC, C/Serrano 113 bis, 28006 Madrid, Spain; 2 University Hospital Ramon y Cajal, 28304 Madrid, Spain; 3 International Breast Cancer Center (IBCC), Pangaea Oncology, Quironsalud Group, 08017 Barcelona, Spain; 4 Medical Scientia Innovation Research (MedSIR), 08018 Barcelona, Spain; 5 Department of Medicine, Faculty of Biomedical and Health Sciences, Universidad Europea de Madrid, 28108 Madrid, Spain

**Keywords:** trastuzumab, pertuzumab, HER2, binding free energy, prodigy, size exclusion chromatography

## Abstract

The binding affinity of trastuzumab and pertuzumab to HER2 has been studied using both experimental and in silico methods. The experiments were conducted using the antibodies in their complete IgG form, as used in clinical therapy, and the extracellular domain of the HER2 protein in solution. This approach provides a precise, reproducible, and reliable view of the interaction between them in physicochemical conditions similar to those found in the tumoral environment. Dynamic light scattering and size exclusion chromatography coupled with tetra detection were utilized to characterize the protein complexes, measure their concentrations, and calculate the equilibrium-free binding energy, ΔG_bind_. In addition, PRODIGY, a QSAR-like model with excellent predictive ability, was employed to obtain in silico ΔG_bind_ estimations. The results obtained indicate that pertuzumab exhibits a slightly higher binding affinity to HER2 than trastuzumab. The difference in binding affinity was explained based on the contribution of the different interfacial contact (IC) descriptors to the ΔG_bind_ value estimated by the PRODIGY model. Furthermore, experiments revealed that the pertuzumab IgG antibody binds preferentially to two HER2 proteins, one per Fab fragment, while trastuzumab mainly forms a monovalent complex. This finding was interpreted based on a geometrical model that identified steric crowding in the trastuzumab–HER2 complex as compared with the pertuzumab–HER2 complex.

## 1. Introduction

Monoclonal antibodies (mAbs) have emerged as highly effective and successful anticancer therapies. In particular, the human epidermal growth factor receptor 2 (HER2) is a widely recognized antigen targeted by various antibodies for the treatment of HER2-overexpressed cancers [1]. Trastuzumab, the most commonly used mAb against HER2, exhibits specificity for domain IV of HER2, a crucial region involved in ligand binding and receptor activation [2]. By binding to this domain, trastuzumab hinders ligand-induced HER2 activation, resulting in the inhibition of downstream signaling pathways vital for cancer cell proliferation and survival. On the other hand, pertuzumab targets domain II of HER2, which is responsible for the receptor’s dimerization and subsequent activation [3]. Pertuzumab’s binding to this region disrupts HER2 dimerization and blocks downstream signaling pathways as well. Comprehending the precise binding sites of both trastuzumab and pertuzumab on HER2 sheds light on the structural and functional aspects of the mAbs–HER2 interaction. This information holds potential for guiding the rational design and optimization of antibody-based therapies aimed at targeting HER2 or other receptors.

Several experimental studies have analyzed the binding affinity of each antibody to HER2. Troise et al. investigated the binding between HER2 and trastuzumab using three different methods (ELISA, SPR, ITC) at room temperature. They estimated the free energy of binding (ΔG_bind_) to be −11.0 ± 3.0 kcal·mol^−1^ [4]. In another study, Lua et al. conducted a binding kinetics analysis of the interaction between the extracellular domain of HER2 and both antibodies, alone or in sequential combination, by using BLItz biosensor technology. The estimated ΔG_bind_ values were −11.1 and −10.5 kcal·mol^−1^ for trastuzumab and pertuzumab antibodies, respectively. In addition, they explored both the whole antibody and only its Fab subdomain. They observed a slightly preferential binding for trastuzumab over pertuzumab in both antibody forms, namely, Fab or the complete IgG form [5]. Furthermore, Spiegelberg et al. determined dissociation constants for HER2/trastuzumab and HER2/pertuzumab complexes using the ligand tracer technique on living cell lines [6]. Their study revealed ΔG_bind_ values of −13.7 and −14.0 kcal·mol^−1^ for the trastuzumab and pertuzumab complexes, respectively, at 310 K.

The molecular structures of the complexes formed by the HER2 extracellular domain and the Fabs of the trastuzumab and pertuzumab antibodies have been investigated using crystallography techniques [2,3]. In addition, cryo-electron microscopy has been employed to examine ternary complexes involving the HER2 antigen with both trastuzumab and pertuzumab Fabs [7]. The availability of such detailed structural information has facilitated several “in-silico” studies of these systems. Fuentes et al. performed MD simulations and MMPBSA calculations to estimate the ΔG_bind_ [8], and their results indicated that trastuzumab exhibits stronger binding affinity compared with pertuzumab. They also suggested the presence of a potential synergistic effect between the two antibodies based on their mutual interaction. Nonetheless, the large size of the protein complexes posed a significant limitation for the simulation models used due to inadequate sampling for ΔG_bind_ evaluation. A recent study by Samsudin et al. reports the successful usage of multiscale approaches to deal with big antigen/antibody systems of the IgM type, which may solve the sampling issue of calculating free energies [9].

In this study, we conducted a biophysical analysis to investigate the binding characteristics of pertuzumab and trastuzumab antibodies in their interaction with the extracellular HER2 domain. In contrast to previous studies, we employed a label-free method to quantitatively characterize macromolecular interactions under physico-chemical conditions similar to those found in the tumoral environment. To the best of our knowledge, this methodology has not yet been tested for this case. Specifically, we employed Size Exclusion Chromatography (SEC) to examine the binding process of each monoclonal antibody to HER2. In our previous studies, SEC has been successfully applied to analyze the hydrodynamic properties of aqueous solutions containing an extracellular HER2 domain, trastuzumab, and their complexes [10]. It is noteworthy that the experimental design in this study was based on a solution-phase approach that considered the entire IgG antibody as it is used in clinical therapy. This approach is known to yield more realistic results compared with protocols that rely on chip antibody immobilization techniques [11]. 

Our in silico approach begins with the crystallographic structures to provide atomistic details of the intermolecular interactions between the antibodies and the antigen. Specifically, we adopted the protein–protein binding model proposed by Vangone et al. [12] to analyze each type of interaction and its individual contribution to the overall binding affinity. This quantitative structure activity relationship (QSAR) model correlates with structural descriptors of the protein complexes that specifically pertain to their interaction within the binding region. To create the model, the authors used a curated database of experimental data on protein complexes for which crystallographic resolved structures were available, resulting in correlations with excellent predictive performance.

Another added value of this approach is its ability to provide a straightforward interpretation of the structural descriptors that contribute to the model, including residue–residue interaction types (e.g., charged–charged, charged–polar, polar–apolar, etc.) and non-interacting molecular surfaces. It is worth noting that we opted against using molecular simulation approaches to calculate ΔG_bind_ given the large size of the systems. Such simulations would require significant computational resources to achieve adequate sampling and converged values. We used short MD simulations starting from the crystallographic structures to provide the QSAR modeling with a larger set of equilibrated structures to obtain robust statistical measurements of the range error in the estimated ΔG_bind_ values.

## 2. Results

### 2.1. Experimental Findings

**Figure 1:**
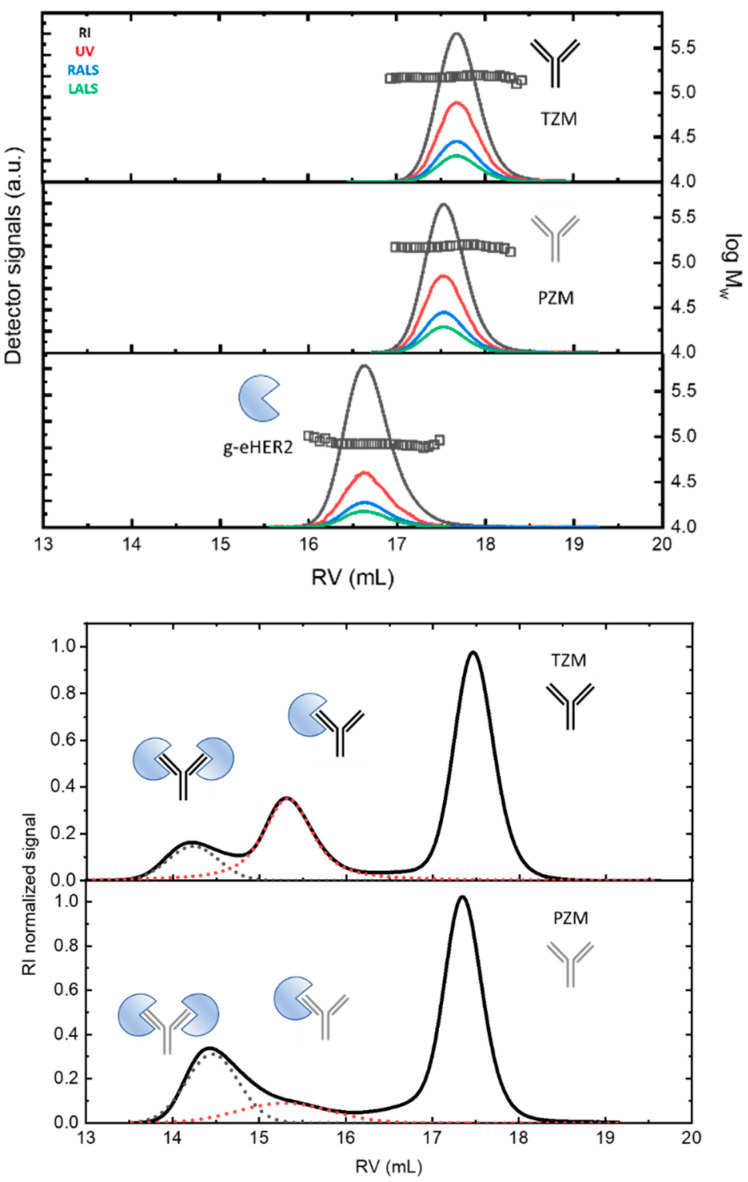
(**Up**) SEC/tetradetection profiles of the mAbs, trastuzumab (TZM) and pertuzumab (PZM), and the antigen, g-eHER2. The lines represent the profiles obtained from the different detectors. The symbols represent the measured absolute molecular masses. (**Down**) SEC/tetradetection profiles of g-eHER2/TZM and g-eHER2/PZM mixtures. The dashed lines represent the deconvolution of complex 1 and 2 signals to determine the corresponding concentrations.

The SEC profiles of the samples at c~0.3 mg·mL^−1^ (for g-eHER2) and c~1.0 mg·mL^−1^ (for mAbs), obtained from the different detectors, are envisaged in Figure 1 (up).

Both mAbs show a similar profile at around a retention volume of 17.5–17.6 mL. Absolute molecular masses of 146.0–147.0 kDa were measured (symbols). The observed molecular mass values agree with the typical molecular size for antibodies of the IgG type (see Table 4 in Section 4). The differences found among the values taken after five independent measurements were lower than 2%. g-eHER2 elutes around 16.6 mL, having a measured absolute molecular mass of 86.4 kDa. The values of [η] and r_h_ were also obtained for all the systems under study (see Table 4). The expected values for both hydrodynamic properties were obtained for mAbs and g-eHER2 [10,13,14].

We extracted the experimental values of the absorption coefficient dA/dc from the UV detector by matching the protein concentration with the value obtained from the refractive index (RI) detector. The values obtained for dA/dc for mAbs and g-eHER2 samples are listed in Table 4. Trastuzumab (1.38 ± 0.01 mL·g^−1^) shows a higher value of dA/dc than pertuzumab (1.29 ± 0.01 mL·g^−1^). The experimental variability was lower than 1% among five independent measurements in each case. Both mAbs show a high degree of chemical similarity. There were subtle differences in the amounts of tyrosine (Tyr), tryptophan (Trp), and phenylalanine (Phe) residues. These differences had an impact on the dA/dc measured using UV spectroscopy. The extinction coefficient at 280 nm was unique for each protein, depending on the number of aromatic residues (Tyr, Trp, and Phe), but it could also be affected by the solvent, the temperature, and the pH. We used the Protein Calculator Resource (http://protcalc.sourceforge.net/, accessed on 22 July 2023) to calculate dA/dc for the samples under study using the corresponding amino acid sequence. The extinction or absorption coefficients in water were estimated in this case using the method of Gill and von Hippel at 280 nm [15]. The results obtained match with the experiments.

In Figure 1 (down), the RI profiles obtained for the 1:3 antigen–antibody solutions are shown. The peaks appearing at the lowest retention volumes indicate the formation of complexes between both the g-eHER2 receptor and the mAbs. For the HER2/trastuzumab mixture, two peaks at retention volumes of 15.4 mL and 14.1 mL are clearly resolved, with molecular weights of 234 and 320 kDa, respectively. These values agree with the formation of both a heterodimer (HER2/trastuzumab or complex 1) and heterotrimer (HER2/trastuzumab/HER2 or complex 2). The deconvolution of the signals allows one to determine the total concentration, and that corresponding to both complexes 1 and 2. The data yield a proportion of 0.31 C_T_ for complex 1 and 0.10 C_T_ for complex 2, with C_T_ being the total concentration of protein measured (Table 5 in Section 4). In the conditions used in this study, the highest proportion of complex obtained in this case corresponds to the heterodimer. In the case of the HER2/pertuzumab mixture, two elution peaks were observed, located around very similar values of retention volumes to those obtained in the HER2/trastuzumab mixture. However, the trend was reversed, since it was mostly the heterotrimer (HER2/pertuzumab/HER2) in which the two fabs of the antibody bind to an antigen. In this case, the concentrations were 0.12 C_T_ for the heterodimer and 0.27 C_T_ for the heterotrimer.

From these results, both the total concentration of antigen and antibodies and the equilibrium concentration of complexes and free antigen can be calculated (Table 5). These data allow one to compare the binding affinity of these two antibodies with the g-eHER2 antigen in aqueous solutions at 309 K. The main question here relates to the antibody valence. Trastuzumab and pertuzumab molecules have two binding sites (Fab domains) which are able to interact with the g-eHER2. In fact, two different complexes are clearly observed in Figure 1 for both systems. In this specific case, we may use the Goldberg’s equation developed for bivalent antibodies [16,17].

(1) $$K=\frac{fM_{a}p}{4c_{a}\left(1-p\right)\left(1-p\frac{fc_{g}M_{a}}{2c_{a}M_{g}}\right)}$$

where c_a_ and c_g_ are total antibody and antigen concentration in g·L^−1^, M_a_ and M_g_ are the molecular mass of antibody and antigen, f is the number of binding sites in the antibody, and p is the fraction of the reacted antigen sites that can be obtained from:

(2) $$c_{ag}=c_{g}{\left(1-p\right)}^{f}$$

where c_ag_ is the free antigen concentration in g·L^−1^. The higher the affinity of the antibody, the higher its equilibrium constant. This may be expressed by means of the equation:

(3) $$-\ln K_{a}=\frac{\Delta G}{RT}$$

where ΔG is the change in free energy, R is the universal gas constant (=1.987 cal·K^−1^·mol^−1^), and T is the absolute temperature (309 K). To convert K_a_ to the SI units of energy, K_a_ must be expressed in [mole fraction]^−1^ rather than in units of M^−1^ [7]. The values obtained for ΔG from Equation (3) are listed in Table 1.

### 2.2. In Silico Binding

In our study, we used the PRODIGY webserver to evaluate the different terms in Equation (5) in Section 4.2.2 for each structure equilibrated through MD simulations. For each system, namely HER2/trastuzumab and HER2/pertuzumab, we uploaded three replicas containing 20 snapshots each. The estimated ΔG_bind_ values were then averaged over the last 20 snapshots for each system and are collected in Table 2. Further details regarding the different contributions accounted for the estimation of ΔG_bind_ can be found in the Supplementary Materials.

Based on the results shown in Table 2, the PRODIGY QSAR equation predicts that pertuzumab is a marginally better binder than trastuzumab. This finding is also supported by the crystallographic structures submitted to the PRODIGY protocol, which yielded lower ΔG_bind_ values for pertuzumab compared with trastuzumab (−10.3 kcal·mol^−1^ versus −12.0 kcal·mol^−1^). Additionally, the pertuzumab complex exhibited a slightly larger number of ICs, which contributed the most to QSAR Equation (5).

Table 3 shows the number of ICs per property and the % of NIS percentage per property for the crystallographic structures, as well as the corresponding averages for the structures obtained using MD simulations. It is evident that the ICs involving charged–polar and ICs polar–polar interactions were quite similar in the HER2/trastuzumab and HER2/pertuzumab complexes. The most remarkable differences were found in the ICs _charged/apolar_ and ICs _polar/apolar_, which were larger in the HER2/pertuzumab case, giving rise to a more favorable binding energy. The NIS percentage contribution was, however, rather similar for both systems.

## 3. Discussion

### 3.1. Binding Affinity

The experimental methodology employed in this study provides a reliable and unambiguous determination of antigen–antibody binding energies using complete mAbs in aqueous solutions. The resulting experimental ΔG_bind_ values are in excellent agreement with the results obtained through the chosen “in-silico” approach. This consistency further confirms the outstanding performance of the QSAR model, which, in turn, was derived from carefully curated experimental data including some antigen–antibody complexes [12].

**Figure 2:**
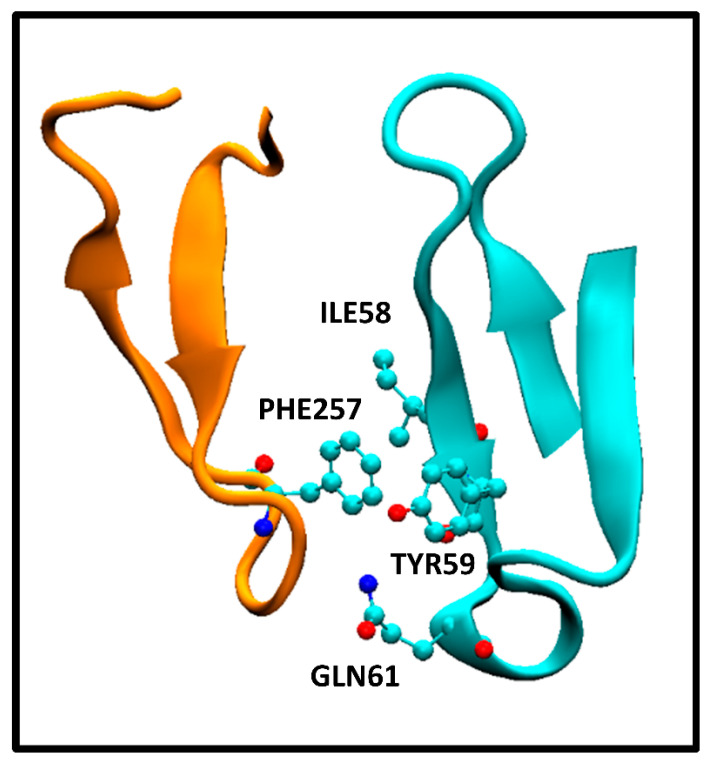
Interaction between HER2 extracellular domain PHE257 and pertuzumab Fab heavy chain variable domain ILE58, TYR59, and GLN61 residues. The specific amino acids are in a ball and stick representation. HER2 and pertuzumab Fab fragments are orange and cyan cartoon representations, respectively.

It is interesting to note the discriminatory ability of the ICs _charged/apolar_ and ICs _polar/apolar_ descriptors in QSAR Equation (5). In principle, it may seem counterintuitive that polar–apolar and charged–apolar interactions contribute to a decrease in ΔG_bind_ value. However, our observations in the antigen–antibody systems studied here reveal that the interaction between apolar and polar/charged amino acids is a consequence of the close proximity of residues between antibody and antigen, as well as the arrangement of polar, apolar, and charged residues in each protein ternary structure. To illustrate this, Figure 2 displays the interaction between PHE257 residue of the extracellular HER2 domain and ILE58, TYR59, and GLN61 residues of the pertuzumab heavy chain Fab.

This corresponds to the crystallographic structure of the HER2/pertuzumab complex (PDB Code: 1S78) analyzed using the PRODIGY protocol. In this structure, three contacts can be observed, namely, an apolar/polar interaction between one GLN61 and PHE257 and two apolar/apolar interactions between ILE58-TYR59 and PHE257. The former one contributes to ICs_polar/apolar_, while the latter two interactions contribute to ICs_apolar/apolar_. These interactions form part of a network of contacts between amino acids from both the antibody and antigen, resulting in a tightly bound complex. The presence of those polar/apolar and charged/apolar interactions correlates with this enhanced binding observed, as the PRODIGY QSAR equation. In contrast, the HER2/trastuzumab complex comprises a higher number of ICs_apolar/apolar_ compared with the HER2/pertuzumab complex. However, these specific interactions were not considered in the QSAR model, although they contribute to the Buried Surface Area (BSA). Consequently, the BSA values reported for the crystallographic structures indicate a larger value for the HER2/trastuzumab complex (1350 Å^2^) compared with HER2/pertuzumab complex (1210 Å^2^). Vangone et al. showed the superior performance of the model described in Equation (5) compared with models that solely rely on BSA estimations [12].

The PRODIGY QSAR model was derived with a solid chemometric protocol using protein–protein complex structures obtained from high-resolution crystallographic data and a curated set of experimental determination of ΔG_bind_. The glycosylation issue was not taken into account explicitly in the QSAR equation, where only interactions between protein amino acids were counted. Probably, glycosylation around the binding site would weaken protein–protein association and, consequently, causes a decrease in the IC numbers in Equation (5).

### 3.2. Antigen–Antibody Complexes’ Stoichiometry

In this work, we have experimentally shown that the IgG form of pertuzumab can bind two HER2 antigens, one per available Fab, more easily than trastuzumab (Figure 1). To provide a possible explanation for this observation, we have built purely geometric models for the complete IgG form of the antibodies with two molecules of the extracellular HER2 domain bound to each Fab fragment.

**Figure 3:**
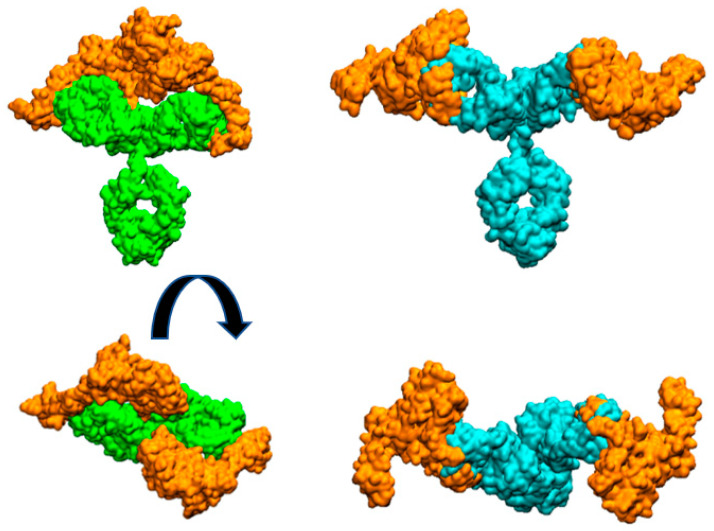
Molecular surfaces showing the relative disposition of whole mAbs with two HER2 extracellular domain proteins (orange color) bound to each Fab. Pertuzumab is colored cyan and trastuzumab is colored green. Figures at bottom are perpendicular views of the upper ones.

Figure 3 illustrates the arrangement of both HER2 extracellular domains bound to the two antibody Fabs. It is evident that trastuzumab may experience more steric hindrance than pertuzumab when accommodating two HER2 antigens on the same IgG antibody. This observation can account for the preferential 2:1 stoichiometry observed in the pertuzumab complex. A similar explanation was given for IgM antibody forms by Samsudin et al. [9].

It is worth noting that we previously studied the structure of the trastuzumab antibody bound to one and two HER2 extracellular domains [10,13]. In these works, we analyzed its hydrodynamic properties. Upon revisiting the simulated systems and examining any potential interferences between both HER2 proteins in the 2:1 complex, we have not found any significant steric hindrance between both HER2 proteins.

**Figure 4:**
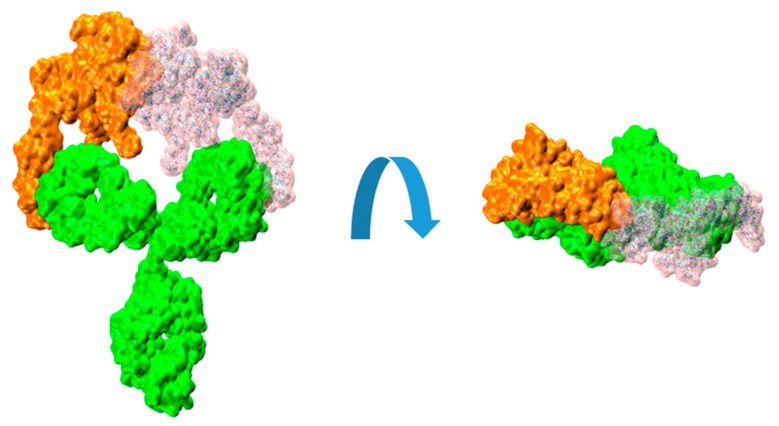
Molecular surfaces showing the relative disposition of trastuzumab IgG (green color) with a bound HER2 extracellular domain proteins (orange color) and another HER2 (transparent pink color) fitted on the available Fab, as described in the text.

It can be illustrative to consider the sequential binding of a second HER2 unit to a HER2/trastuzumab complex, where a first HER2 protein has already been bound. In the present work, we used those simulations to explore whether the mobility of the HER2–trastuzumab Fab bound complex, relative to the unbound Fab within the IgG molecule, could potentially hinder the binding of a second HER2 protein. Video S1 in the Supplementary Material shows a recording from an MD simulation demonstrating the flexibility of the Fab arms in the IgG structure when one of them is bound to an HER2 protein. Taking this flexibility into consideration, we sequentially fitted another HER2 protein in the available Fab. This was repeated for several structures of the 1:1 complex obtained from the MD simulations performed in our previous work. For the fitting procedure, we used the substructure formed by the bound HER2 and the Fab light chain. That set was superimposed on the light chain of the available Fab, resulting in a virtual 2:1 complex. The result can be visualized in Figure 4.

The resulting root mean square distance (RMSD) between the backbone atoms of the two light-chain fragments was around 2 Å. The observed collapse between the two HER2 substructures is clearly evident, enhanced by the interpenetration of their surfaces. Although this analysis is a rigid procedure showed for illustrative purposes, it can effectively convey the difficulties associated with the binding of a second HER2 protein to the HER2/trastuzumab 1:1 complex to form the 2:1 antigen/antibody configuration. 

Higher avidity, which is associated with an antibody interacting with an overexpressed cellular surface receptor, may be expected to exhibit enhanced efficiency against its antigen [18]. Moreover, the combination of improved HER2 avidity and the slightly higher binding affinity of pertuzumab compared with trastuzumab makes it an appealing monoclonal antibody for therapies involving the transportation of potent cytotoxic agents to cancer cells overexpressing target receptors, such as in ADC (Antibody Drug Conjugate) treatments. 

## 4. Materials and Methods

### 4.1. Experimental Details

#### 4.1.1. Samples

The mAbs pertuzumab Perjeta^©^ and trastuzumab Herceptin^©^ (stock solution at 30 and 21 mg⋅mL^−1^, respectively) were kindly provided by one of us (JC) from Hospital Ramón y Cajal (Madrid, Spain). The glycosylated HER2 receptor ectodomain tagged with a 10-length polyhistidine peptide on the C-terminal (g-eHER2) was purchased from Sino Biological, Inc. (Beijing, China). Desalting and buffer exchange were carried out with centrifugal concentrators (Amicon Ultra−0.5 mL, Merck Millipore, Billerica, MA, USA). All solutions were filtered (Millex-GV 0.22 μm, Merck Millipore, Billerica, MA, USA) and stored in 20 mM Tris-HCl pH 7.5, 150 mM NaCl to subsequent analysis and to prepare g-eHER2/mAbs mixtures at the selected molar ratio 1:3. Water for all buffers and dilution was obtained from Milli-Q water purification system (Merck Millipore, Billerica, MA, USA). The initial concentrations of g-eHER2 and mAbs in the stock solutions were 0.3 and 1.0 mg⋅mL^−1^, respectively. Total initial concentration of protein in the mixtures was maintained nearly constant, around 0.90 mg⋅mL^−1^. The g-eHER2 concentration selected was low enough to avoid the formation of homodimer, reported for concentrations around 1 mg⋅mL^−1^ in his-tagged g-eHER2 samples [10]. Solutions were mixed at 300 r.p.m. and T = 309 K for 30 min in an Eppendorf Thermomixer (Eppendorf, Enfield, CT, USA).

#### 4.1.2. Basic Hydrodynamic Characterization of e-HER2 Receptor and mAbs

Dynamic light scattering (DLS) electric field correlations were obtained for the g-eHER2 and mAbs solutions prepared as indicated above, using the Zetasizer Nano ZS (Malvern Instruments, Worcestershire, UK) at T = 309 K, equipped with disposable cuvettes (Malvern Instruments ZEN0040). The readers are referred to previous publications for the experimental details [10,13]. This technique allowed us to extract the diffusion coefficient, D_s_, from the autocorrelation function. Hydrodynamic size, r_h_, was obtained from the results obtained for D_s_, the diffusion coefficient at infinite dilution, using the Stokes–Einstein equation:

(4) $$D_{s}=\frac{k_{B}T}{6\pi \eta r_{h}}$$

with k_B_, the Boltzmann constant, and η, the buffer viscosity, at T = 309 K. The results obtained for D_s_ and r_h_ are shown in Table 4.

#### 4.1.3. Molecular Weight and Concentration of g-eHER2 Receptor, mAbs, and Complexes

Conventional size exclusion chromatography (SEC) relies on a single concentration detector and calibration standards to determine relative molecular weight, which can lead to imprecise results for proteins, mAbs, and complexes with non-globular shapes. In this study, we used an SEC system employing multiple detectors to overcome these limitations. The determination of the molecular weight, M_w_, the concentration of the g-eHER2 receptor, the mAbs, and the HER2/mAbs complexes in solution was carried out by means of size exclusion chromatography (SEC) coupled with tetradetection using a GPCmax-TDA system (Malvern Instruments, Worcestershire, UK) at T = 309 K. A GE Superose™ 300 column (GE Healthcare, Buckinghamshire, UK) was equilibrated with a buffer composed of 20 mM Tris pH 7.5, 150 mM NaCl. Our system comprises two concentration detectors (refractive index—RI and ultraviolet—UV), a light scattering detector (LS), and a viscometer (VIS). The LS detector enabled direct measurement of absolute Mw, eliminating the need for calibration standards of different sizes and molecular weights. Additionally, the VIS detector provided valuable information on intrinsic viscosity, [η], and molecular density. Our system was calibrated using a single standard, bovine serum albumin (BSA), to derive the characteristic constant for each detector. This procedure offers enhanced characterization capabilities, enabling precise molecular weight determination and structural insights without relying on standard calibration. Notwithstanding, we validated the accuracy of the calculated constants with BSA by eluting different standard proteins, including thyroglobulin, ferritin, aldolase, conalbumin, and ovalbumin, as it is usually done in other studies [19]. In total, 100 μL of sample of concentrations 0.3 mg·mL^−1^ (g-HER2), 1 mg·mL^−1^ (mAbs), and those indicated in Table 2 for the mixtures (0.9 mg·mL^−1^), were injected into the SEC column and eluted with the equilibration buffer at a flow rate of 0.5 mL·min^−1^ (see Table 1). Elution profiles were followed by a UV-photo diode array (UV-PDA), a differential refractometer (RI), a 7° low-angle light-scattering detector (LALS), and a 90° right-angle light-scattering detector (RALS). Bovine serum albumin (BSA) was used as standard reference protein of known molecular weight, concentration, and refractive index increment (dn/dc = 0.185 mL·g^−1^). The OmniSEC 4.8 software program was used for the data acquisition and analysis, including the absolute molecular weight, M_w_, the intrinsic viscosity, [η], the specific absorption coefficient, dA/dc (Table 4), and, finally, the concentration, c, of each sample was determined (Table 5).

### 4.2. In Silico Methods

#### 4.2.1. Molecular Dynamics Simulations

The starting structures for the extracellular HER2 domain and the Fabs of the mAbs trastuzumab and pertuzumab were extracted from the protein data bank (codes:1N8Z for the HER2/trastuzumab [2]. Fab complex and 1S78 for the HER2/pertuzumab [3]. Fab complex). Missing residues in both structures were filled using the protein loop tools available in the Discovery Studio package [20]. The Molecular Mechanics models selected for the simulation consist of the force field ff14SB [21] for proteins, as implemented in the Amber 16 suite of programs [22]. This model contains the adequate parameters for the systems considered in this work. The models built for each protein complex described in the previous point were subjected to the same refinement protocol described in previous works [10,13].

#### 4.2.2. PRODIGY QSAR Model

A subset of 20 snapshots at 1 ns interval corresponding to the final 20 ns of the NVT simulations for each system was submitted to the PRODIGY webserver for binding energy evaluation [23]. The QSAR equation used to evaluate binding affinity was that described by Vangone et al. [12]:

(5) $$\Delta G_{predicted}=-0.09459{IC}_{\frac{charged}{charged}}-0.10007{IC}_{\frac{charged}{apolar}}+0.19577{IC}_{\frac{polar}{polar}}-\;0.22671{IC}_{\frac{polar}{apolar}}+0.18681\%{NIS}_{apolar}+0.3810\%{NIS}_{charged}-15.9433$$

where IC descriptors correspond to nature-dependent antigen–antibody interfacial contacts and NIS is the percentage of non-interacting surface (see Ref. [24] for details). Two residues are defined in contact if any of their heavy atoms are within a distance of 5.5 Å. The final ΔG values were averaged over the results obtained for each structural set submitted to the PRODIGY webserver. The IC descriptor is the number of contacts between residues of the interacting proteins according to the amino acid types. The amino acids were classified as: charged (E, D, K, R), polar (C, H, N, Q, S, T, W), and apolar (A, F, G, I, L, V, M, P, Y). The subscript in each of these IC descriptors shown in Equation (2) refers to the residue classes of the interacting proteins. For example, ${IC}_{\frac{charged}{apolar}}$ is the number of contacts between charged residues of one protein and apolar residues of the other.

#### 4.2.3. Models for Antigen–Antibody Complexes’ Stoichiometry

The models for the complete IgG form of trastuzumab as well as its 1:1 and 1:2 complexes were obtained from our previous publication [13]. The model for pertuzumab was built using trastuzumab as a template and using the sequence and structure alignment tools available in Discovery Studio package [20] along with the crystallographic structures of the HER2 extracellular domain- pertuzumab Fab complexes [3,7].

## 5. Conclusions

In this study, a combined experimental and in silico analysis was conducted to investigate the binding process of trastuzumab and pertuzumab mAbs to the extracellular HER2 domain. The experimental setup employed size exclusion chromatography to separate protein complexes by size, enabling the determination of ΔG_bind_ via the differentiation between different stoichiometries and the determination of the concentration of each species_._ The binding free energy obtained from this label-free experimental methodology experiment is in excellent agreement with the in silico results, showing that pertuzumab is a marginally better binder than trastuzumab. In this respect, the QSAR model PRODIGY employed in the in-silico analysis revealed that the pertuzumab complex presents more interfacial contacts (ICs) than the trastuzumab complex. More specifically, the pertuzumab complex presents a higher number of ICs of the charged/apolar and polar/apolar types, which significantly contribute to reduce the ΔG_bind_ in the QSAR model. These favorable contacts observed can be attributed to the local approximations of charged or polar residues to apolar amino acids, constituting an enhanced interaction network. Furthermore, the study reveals two types of associations for these antibodies, namely, the intrinsic association (affinity) for the monovalent interaction and functional affinity (avidity) for the divalent interaction. The antibody IgG forms used in the experiments showed a superior avidity, the preferential divalent functionality, of pertuzumab over trastuzumab. This preference may reside in the close proximity between both antigens bound to the two available Fab arms of the IgG trastuzumab antibody. It should be highlighted that the results of this study were obtained from unmodified and label-free mAbs in solution, which offers interesting advantages. Firstly, it avoids any possible alteration of the mAbs structure or function that may occur during modification or labeling. Secondly, it minimizes the risk of interference of the modifications in the interaction between the mAbs and HER2. Additionally, the use of unmodified and label-free mAbs allows experiments to be performed under physico-chemical conditions similar to those found in the tumoral environment, better reflecting the conditions under which interactions occur in the human body.

Several studies have explored the interaction between HER2 and monoclonal antibodies (trastuzumab and pertuzumab). The references are included in the list of citations for this work. The majority of these studies have focused on the clinical aspects and practical relevance of the HER2–mAb interaction. However, there has been a lack of quantitative studies conducted in solution, such as the one presented here. In this study, we have employed a combined approach of experiments and simulations to gain deeper insights into this interaction.

Regarding the simulations, the quantitative structure–activity relationship (QSAR) approach has been demonstrated to be highly effective in predicting binding energies, in agreement with experimental data. This computational method enhances our understanding of the binding process and provides valuable insights into the molecular interactions involved. Furthermore, the experiments and simulations conducted in this study have revealed distinct behaviors of trastuzumab and pertuzumab. Pertuzumab preferentially binds to two HER2 proteins, forming a bivalent complex. In contrast, trastuzumab primarily forms a monovalent complex. This disparity in binding modes between the two antibodies holds intriguing implications that require further exploration.

By combining both experimental and computational approaches, this study offers a comprehensive understanding of the HER2–mAb interaction, shedding light on the underlying mechanisms and highlighting the differential behavior of trastuzumab and pertuzumab. These findings contribute to the growing body of knowledge in this field and pave the way for potential therapeutic applications.

## Figures and Tables

**Table 1:** Equilibrium parameters and determination of the free energy, ΔG, of antigen–antibody interactions.

Sample	K_a_ (Mole Fraction^−1^) × 10^−7^ s.d. < 2%	ΔG (kcal·mol^−1^) s.d. < 2%
HER2/Trastuzumab	2.49	−10.4
HER2/Pertuzumab	3.17	−10.6

**Table 2:** ΔG_bind_ values estimated at T = 309 K using the PRODIGY protocol.

System	ΔG_bind_ (kcal·mol^−1^)	
	Crystal Structures	MD Structures
HER2/Trastuzumab	−10.3	−11.0 ± 0.8
HER2/Pertuzumab	−12.0	−12.2 ± 0.9

**Table 3:** Percentage of nature-dependent antigen–antibody interfacial contacts (ICs) and that of non-interacting surface (NIS) calculated with the PRODIGY protocol on crystallographic structures and structures extracted from MD simulations (averaged values over 20 structures, see text for details). The number of ICs per property is also shown.

QSAR Component	HER2/Trastuzumab		HER2/Pertuzumab	
	Crystal	MD	Crystal	MD
NIS charged (%)	21	20 ± 1	21	20 ± 1
NIS apolar (%)	36	38 ± 1	37	38 ± 1
Number of ICs per property				
ICs charged–charged	7	8 ± 4	3	3 ± 1
ICs charged–polar	7	12 ± 5	13	12 ± 2
ICs charged–apolar	16	20 ± 5	23	22 ± 4
ICs polar–polar	2	5 ± 3	10	6 ± 1
ICs polar–apolar	8	12 ± 3	24	20 ± 3
ICs apolar–apolar	21	29 ± 2	17	17 ± 4

**Table 4:** Hydrodynamic and molecular properties of g-eHER2, mAbs, and complexes present in the highest proportions, obtained from DLS, SEC, and tetradetection.

Sample	M_w_ (kDa) s.d. < 2%	[η] (cm^3^·g^−1^) s.d. ± 0.2	dA/dc (mLg^−1^cm^−1^) s.d. ± 0.02	D_s_ × 10^7^ (μm^2^·s^−1^) s.d. ± 0.2	r_h_ (nm) s.d. ± 0.1
Trastuzumab—TZM	147.0	6.5	1.38 (1.40)	58.1	5.5
Pertuzumab—PZM	146.4	6.4	1.29 (1.36)	58.2	5.5
g-eHER2	86.4	6.5	0.90 (0.90)	44.6 *	4.7 *
HER2/TZM	234.0	7.4 *	n.d.	31.6 *	6.8 *
HER2/PZM/HER2	320.0	8.6	n.d.	n.d.	7.4

* Obtained from refs. [9,12] at T = 293 K. The values in parentheses were calculated at 280 nm^−1^ using the protein Calculator v.3.4 tool. n.d. means not determined.

**Table 5:** Initial and equilibrium molar concentrations (μM) of the systems studied.

	Initial Concentrations ^a^ s.d. < 2%		Equilibrium Concentrations s.d. < 2%			
**Sample**	**mAb**	**g-eHER2**	**Complex 1**	**Complex 2**	**Free mAb**	**Free g-eHER2**
HER2/TZM	4.78	1.46	1.12	0.26	3.37	0.08
HER2/PZM	4.76	1.16	0.42	0.68	3.36	0.04

^a^ The initial concentrations were ~0.7 mg·mL^−1^ for mAbs and ~0.11 for g-eHER2.

## Data Availability

All data available upon request to the corresponding author.

## Supplementary Materials

The following supporting information can be downloaded at: https://www.mdpi.com/article/10.3390/ijms241512031/s1.
