# Supplementary material for: Binding Affinity of Trastuzumab and Pertuzumab Monoclonal Antibodies to Extracellular HER2 Domain
Source: Int J Mol Sci. 2023 Jul 27;24(15):12031. doi: 10.3390/ijms241512031 (PMC10418494; doi:10.3390/ijms241512031)
Supplement: Supplementary file 1 [file ijms-24-12031-s001.zip › pertuzumab_HER2_PRODIGY_result_frame_40.pdf]

Description of the intermolecular contacts between HER2 extracellular domain and Pertuzumab Fab.

[+] No. of intermolecular contacts: 80

[+] No. of charged-charged contacts: 3

[+] No. of charged-polar contacts: 12

[+] No. of charged-apolar contacts: 25

[+] No. of polar-polar contacts: 5

[+] No. of apolar-polar contacts: 22

[+] No. of apolar-apolar contacts: 13

[+] Percentage of apolar NIS residues: 38.54

[+] Percentage of charged NIS residues: 20.41

[++] Predicted binding affinity (kcal.mol-1):   -12.7

[++] Predicted dissociation constant (M) at 25.0˚C: 4.7e-10

Listing of residue-residue interactions. Chain A corresponds to HER2 extracellular domain. Chain B corresponds to pertuzumab Fab.

VAL 286 A ASP 867 B

THR 268 A THR 866 B

VAL 286 A THR 866 B

LEU 295 A TYR 868 B

VAL 286 A ASN 890 B

THR 268 A ARG 910 B

HIS 245 A VAL 908 B

LEU 244 A ARG 910 B

LYS 311 A SER 939 B

PRO 294 A TYR 868 B

HIS 296 A TYR 671 B

LEU 244 A SER 907 B

VAL 286 A ASN 888 B

VAL 286 A SER 891 B

LYS 311 A GLY 937 B

LEU 295 A ARG 934 B

THR 290 A ASP 867 B

SER 313 A TYR 675 B

THR 284 A ASP 867 B

LYS 311 A TYR 941 B

HIS 296 A LEU 936 B

LYS 128 A SER 911 B

LYS 314 A ARG 676 B

LYS 314 A TYR 675 B

PRO 315 A TYR 675 B

PHE 257 A SER 894 B

LEU 244 A ASP 909 B

GLY 287 A ASN 888 B

LYS 311 A PRO 938 B

SER	288	A	ASN	890	B
LEU	244	A	VAL	908	B
PHE	257	A	TYR	716	B
PHE	269	A	ARG	910	B
HIS	245	A	ASN	890	B
THR	284	A	THR	866	B
ASP	285	A	ASN	888	B
VAL	286	A	THR	869	B
PRO	247	A	SER	891	B
PHE	257	A	LYS	901	B
PHE	236	A	SER	911	B
GLY	270	A	ARG	910	B
TYR	252	A	ILE	895	B
HIS	245	A	PRO	889	B
VAL	286	A	TYR	868	B
PRO	315	A	ARG	676	B
ASP	285	A	PRO	938	B
HIS	245	A	GLY	892	B
PHE	257	A	TYR	896	B
THR	254	A	LYS	901	B
PRO	294	A	ASP	867	B
LYS	314	A	TYR	671	B
GLY	287	A	THR	866	B
SER	288	A	THR	866	B
LYS	311	A	LEU	936	B
HIS	245	A	SER	891	B
SER	313	A	TYR	671	B
CYS	289	A	ASP	867	B
ASN	297	A	PRO	938	B
SER	272	A	ARG	910	B
CYS	246	A	ASN	890	B

HIS	296	A	GLY	937	B
PRO	315	A	THR	678	B
PRO	315	A	TYR	671	B
CYS	246	A	SER	891	B
HIS	296	A	SER	939	B
LEU	234	A	SER	911	B
CYS	312	A	TYR	671	B
ALA	248	A	SER	891	B
ASN	297	A	GLY	937	B
CYS	246	A	ARG	910	B
HIS	245	A	ASP	909	B
SER	288	A	ASP	867	B
PHE	236	A	ARG	910	B
ASP	255	A	LYS	901	B
PRO	315	A	TYR	677	B
THR	268	A	ASN	890	B
LEU	244	A	ASN	890	B
GLY	287	A	ASN	890	B
VAL	286	A	PRO	889	B
PHE	257	A	ILE	895	B
