# Supplementary material for: Binding Affinity of Trastuzumab and Pertuzumab Monoclonal Antibodies to Extracellular HER2 Domain
Source: Int J Mol Sci. 2023 Jul 27;24(15):12031. doi: 10.3390/ijms241512031 (PMC10418494; doi:10.3390/ijms241512031)
Supplement: Supplementary file 1 [file ijms-24-12031-s001.zip › trastuzumab_HER2_PRODIGY_result_frame_40.pdf]

Description of the intermolecular contacts between HER2 extracellular domain and Trastuzumab Fab.

[+] No. of intermolecular contacts: 76

[+] No. of charged-charged contacts: 6

[+] No. of charged-polar contacts: 8

[+] No. of charged-apolar contacts: 21

[+] No. of polar-polar contacts: 1

[+] No. of apolar-polar contacts: 9

[+] No. of apolar-apolar contacts: 31

[+] Percentage of apolar NIS residues: 38.72

[+] Percentage of charged NIS residues: 20.43

[++] Predicted binding affinity (kcal.mol-1): -10.4

[++] Predicted dissociation constant (M) at 25.0˚C: 2.3e-08

Listing of residue-residue interactions. Chain A corresponds to HER2 extracellular domain. Chain B corresponds to trastuzumab Fab.

MET	589	A	TYR	671	B
GLU	598	A	TYR	714	B
GLU	303	A	GLN	849	B
TYR	588	A	TYR	671	B
PHE	573	A	TRP	935	B
PRO	572	A	HIS	713	B
ILE	591	A	PHE	675	B
ALA	559	A	ARG	895	B
GLU	303	A	GLY	851	B
LYS	583	A	PHE	940	B
PRO	603	A	PHE	675	B
PRO	590	A	SER	672	B
PHE	573	A	TYR	941	B
LYS	569	A	TYR	714	B
LYS	583	A	GLY	939	B
PRO	571	A	ALA	654	B
ASP	596	A	ASN	652	B
PRO	557	A	ASN	891	B
PRO	572	A	ARG	886	B
MET	589	A	PHE	675	B
TYR	588	A	PHE	675	B
GLU	558	A	ARG	886	B
GLU	558	A	THR	894	B
PRO	571	A	THR	715	B
LEU	586	A	PHE	940	B
PRO	603	A	SER	672	B
PHE	573	A	ARG	886	B
GLU	303	A	PRO	850	B
ASP	570	A	GLY	939	B

TYR	588	A	LEU	676	B
PRO	590	A	TYR	671	B
PRO	557	A	TYR	893	B
ASP	570	A	TYR	941	B
PRO	603	A	THR	653	B
PRO	571	A	TYR	714	B
PRO	584	A	PHE	940	B
PRO	584	A	TYR	671	B
ASP	560	A	ARG	895	B
ALA	535	A	ASP	898	B
ASP	560	A	THR	716	B
ALA	535	A	LYS	901	B
PRO	572	A	THR	716	B
GLU	558	A	TYR	888	B
PRO	571	A	HIS	713	B
ALA	600	A	TYR	714	B
GLN	602	A	THR	653	B
PRO	572	A	TRP	935	B
PHE	573	A	TYR	869	B
PRO	590	A	PHE	675	B
GLN	561	A	ARG	895	B
ASN	534	A	ASP	898	B
LEU	586	A	SER	678	B
ASP	304	A	GLY	851	B
GLU	558	A	TYR	869	B
LYS	583	A	ASP	938	B
PRO	584	A	GLY	939	B
PRO	557	A	TYR	869	B
GLY	556	A	TYR	893	B
ASN	534	A	LYS	901	B
PRO	572	A	THR	715	B

GLU	598	A	ASP	650	B
ASP	560	A	ARG	886	B
LEU	586	A	TYR	677	B
GLU	303	A	GLY	852	B
PRO	572	A	TYR	714	B
LEU	586	A	TYR	671	B
TYR	532	A	ARG	895	B
CYS	601	A	THR	653	B
PRO	572	A	TYR	941	B
PRO	571	A	TYR	941	B
ALA	302	A	GLY	851	B
GLU	598	A	ASN	652	B
GLU	558	A	ARG	895	B
PHE	555	A	TYR	893	B
LEU	586	A	LEU	676	B
GLU	558	A	TYR	893	B
