# Supplementary figures and images for: Binding Affinity of Trastuzumab and Pertuzumab Monoclonal Antibodies to Extracellular HER2 Domain
Source: Int J Mol Sci. 2023 Jul 27;24(15):12031. doi: 10.3390/ijms241512031 (PMC10418494; doi:10.3390/ijms241512031)

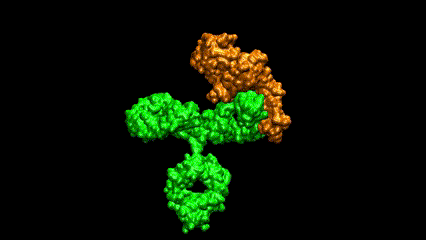

Supplement: Supplementary file 1 [file ijms-24-12031-s001.zip › HER2_trastuzumab.gif]
